# Physiological Mechanisms and Therapeutic Applications of Microneedling: A Narrative Review

**DOI:** 10.7759/cureus.80510

**Published:** 2025-03-13

**Authors:** Lily Tehrani, Michelle Tashjian, Harvey N Mayrovitz

**Affiliations:** 1 Dr. Kiran C. Patel College of Osteopathic Medicine, Nova Southeastern University, Fort Lauderdale, USA; 2 Dr. Kiran C. Patel College of Allopathic Medicine, Nova Southeastern University, Davie, USA

**Keywords:** angiogenesis, ceramides, collagen, drug delivery, elastin, filaggrin, microneedling, percutaneous collagen induction, transepidermal water loss

## Abstract

Microneedling (MN), also known as percutaneous collagen induction therapy, is a minimally invasive dermatologic procedure that stimulates the skin's intrinsic wound repair cascade by creating controlled micro-injuries to the epidermis and dermis using multiple small-sized needles. This review aims to document and discuss the skin's physiological mechanisms activated through the MN process and its therapeutic applications and, where possible, to describe the impacts on changes in the skin's biophysical properties. Three databases, namely, PubMed, Web of Science, and Embase, were searched for relevant peer-reviewed articles published in English between 1990 and 2024. After eliminating duplicate and irrelevant articles, 70 studies were included in this review. The main physiological mechanisms associated with the MN process were collagen and elastin production, angiogenesis, transient increases in skin permeability, and improved epidermal barrier function post-treatment. Therapeutic applications targeted cosmetic improvements, scar healing, and drug delivery. As the wound repair process is initiated, fibroblasts migrate to the wounded area to initiate collagen and elastin production, contributing to the improved firmness and elasticity of the healed epidermis. The micropores created by MN increase skin permeability, allowing hydrophilic water-soluble molecules to transfer across the skin to enhance transdermal drug delivery and absorption. Multiple growth factors are secreted by monocytes upon injury and contribute to collagen production, epithelization, and angiogenesis, which increase epidermal thickness and epidermal barrier enhancement found post-procedure. Additionally, TGFM-1, a cross-linker of the protein filaggrin, and ki67, a marker of cell proliferation, are upregulated following the controlled tissue injury. These upregulated biomarkers contribute to the increase in filaggrin and the improvement of skin barrier function. Ceramides, which help retain moisture and prevent transepidermal water loss, are also increased when MN is combined with a solution containing human adipose tissue stem cell-derived exosomes. The cosmetic applications included improvements in skin texture, wrinkles, and scarring. As a minimally invasive procedure, MN is reported to have a low risk of post-procedural hyperpigmentation, scarring, or other adverse effects.

## Introduction and background

The microneedling (MN) process

MN, also known as percutaneous collagen induction therapy, was introduced in the late 1990s using a device called a dermaroller to improve scars, aging skin, and the transdermal barrier [1]. Dermatologists or other trained workers most often do the procedure as an outpatient procedure, but some devices on the market are advertised for home use as their popularity has steadily increased over the last few years [1]. MN is a process in which multiple small-sized needles are used to cause minor injury to the epidermis and dermis, creating micro-wounds that initiate the body's natural healing cascade to initiate the formation of new collagen in the wounded skin areas, ultimately resulting in an improvement in the skin's condition and appearance [2]. The needle's length can vary depending on the thickness of the skin's dermis. A needle length of 1.5-2 mm is generally used for the routine treatment of acne and other scars, while needle lengths of 0.5 mm or 1 mm are typically used for treating aging skin and wrinkles [3]. The MN process is non-ablative, allowing for the preservation of the epidermis while causing less inflammation and dyspigmentation than other invasive techniques, such as surgical incisions or a CO2 laser treatment [4].

Tissue responses to MN

Once the micro-incisions have been made, multiple inflammatory mediators aid in the three-step process of wound healing. Initially, transforming growth factor (TGF), platelet-derived growth factor (PDGF), and connective tissue activation protein (CTAP) are released from surrounding platelets and neutrophils. These contribute to the production of collagen, elastin, and glycosaminoglycans [5]. These growth factors also produce an intercellular matrix, which provides cell support and communication. Approximately five days after the MN procedure, a fibronectin matrix is formed from an arrangement of β-fibroblasts. These fibroblasts determine the deposition of new collagen that remain in place for 5-7 years, naturally tightening before degrading. The combined protein expression of collagen, glycosaminoglycans, and growth factors ultimately help the skin in dermal regeneration [5].

MN devices

At Home Use 

For home use, most available microneedle devices are adaptations of the Dermaroller (Daejong Medical, Seoul, South Korea). Needle lengths can vary depending on whether the instrument is medically used or used at home. The medical dermaroller is a portable hand-held cylindrical tool that consists of 192 needles organized into eight rows with needle lengths ranging from 0.5 to 1.5 mm and 0.1 mm in diameter [6]. Home care dermarollers have a needle length of less than 0.15 mm, allowing home use for the treatment of fine lines, pore sizes, sebum production, and the transdermal delivery of anti-aging products [6].

In Office Treatment

Another type of MN device is the Dermapen (DermapenWorld, Sydney, New South Wales, Australia). The Dermapen is a portable and electrically powered device that allows for repetitive skin puncturing using a manual stamping-like motion [7]. In a study comparing Dermapen needle lengths and depths of penetration, researchers found that needle lengths of 2.5 mm were more effective in treating atrophic post-acne scars than 1.5 mm needle lengths [8]. In conjunction with the conventional MN principles, newer devices have been developed incorporating additional technology. For instance, light-emitting MN devices incorporate titanium microneedles and light-emitting diodes (LED) to treat wrinkles and scarring [3]. Another approach uses a device known as DermaFrac (Genesis Biosystems, Lewisville, Texas, United States), which combines MN with microdermabrasion, serum infusion, and LED therapy to treat sun-damaged scars, hyperpigmentation, and aging skin [9]. Microneedle fractional radiofrequency (MFRF) is a technique that uses either insulated or non-insulated microneedles to penetrate the skin and heat the superficial and deep dermis with radiofrequency energy to create thermal micro-wounds which then heal using the typical wound healing cascade. In a study analyzing nine participants undergoing six treatments with fractional radiofrequency, researchers found that their patients had no adverse effects and no downtime, as only fractions of the skin were treated at a time [10]. The dermis is minimally damaged during this process as an electronically controlled motor is used to insert the needles into the skin carefully. The papillary and reticular dermis undergo small areas of coagulation, initiating dermal repair. Treatment with MFRF has been reported to help treat scarring, skin texture, and pore size reduction [11]. Additionally, MN with radiofrequency has been reported to improve skin texture and wrinkles [12].

Review Goals

Microneedles traverse the stratum corneum, eliciting controlled skin injury and subsequent regenerative responses. While initially conceived as a conduit for skin regeneration, MN's physiologic and therapeutic implications have gained prominence in dermatology [13]. This review aims to document and discuss the skin's physiological mechanisms activated through the MN process and its therapeutic applications and, where possible, to describe the impacts on the skin's biophysical property changes.

## Review

Methods 

Informative Sources and Search

**Figure 1:**
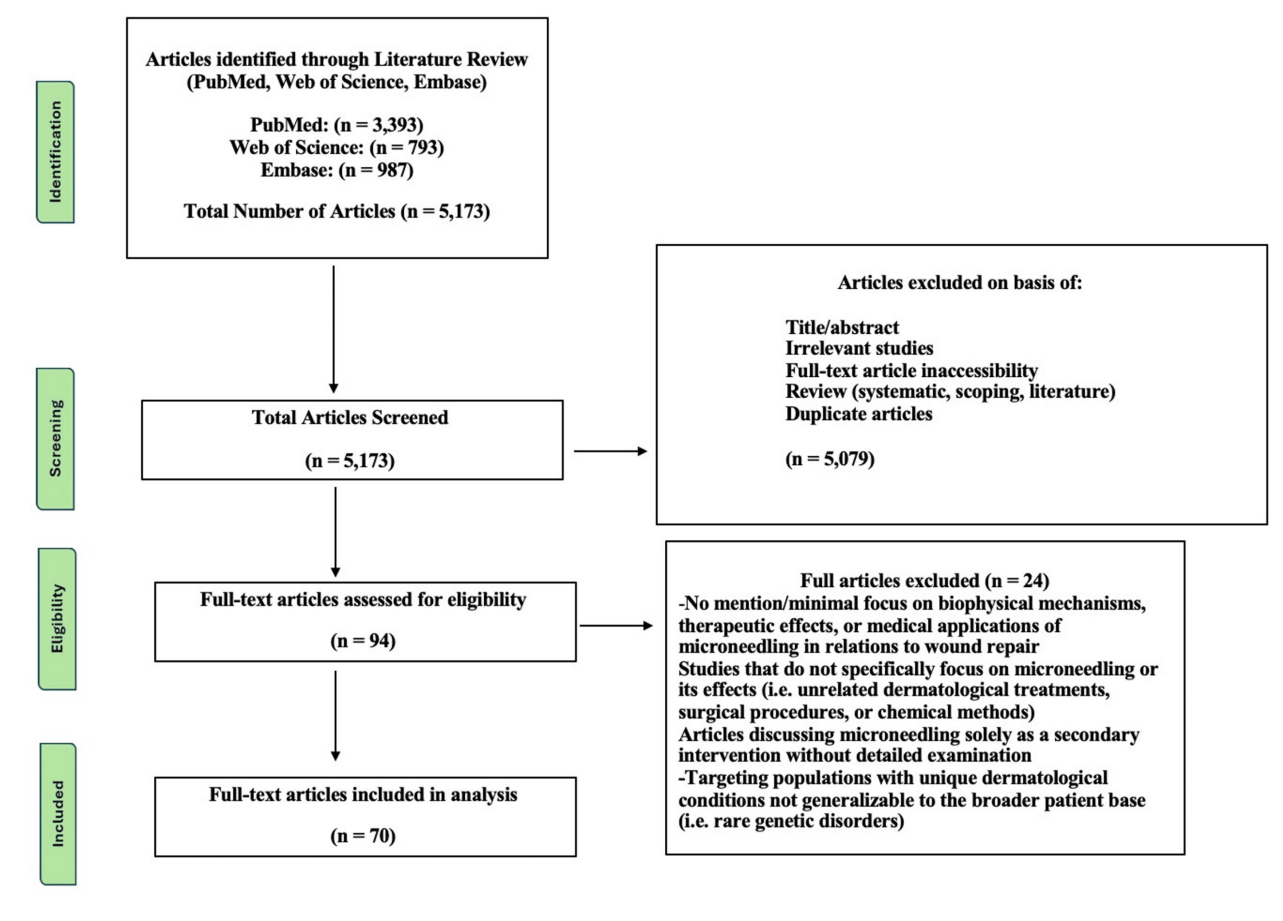
Search procedure

Seventy documents were retrieved from the literature, which discussed the complex, inherent nature of the skin's biophysical mechanisms and how MN affects these processes. Figure 1 depicts the full search sequence with screening and selection in the form of a Preferred Reporting Items for Systematic Reviews and Meta-Analyses (PRISMA)-like diagram [14].

Study Design and Search Strategies 

Three databases (PubMed, Web of Science, and Embase) were searched for full-text peer-reviewed articles written in English and published between 1990 and 2024. Search terms included "microneedling", "transepidermal water loss", "percutaneous collagen induction", "collagen", "elastin", "filaggrin", "ceramides", "angiogenesis", "drug delivery", "TEWL", and "skin hydration". As shown in Figure 1, 5,173 records were identified from the three databases. During the initial screening process, 5,079 articles were excluded, 2,412 duplicates were removed using EndNote 21 (Clarivate, London, United Kingdom), and 2,667 articles were excluded based on title/abstract screening, irrelevance to the study, inaccessibility of full-text articles, or classification as reviews. In the full-text screening process, two reviewers assessed 94 texts. They excluded articles that failed to mention or had a minimal focus on the biophysical mechanisms, therapeutic effects, or medical applications of MN concerning wound repair. Additionally, studies that did not specifically address MN or its effects (e.g., unrelated dermatological treatments, surgical procedures, or chemical methods), articles discussing MN solely as a secondary intervention, and studies targeting populations with unique dermatological conditions that were not generalizable to the public (e.g., rare genetic disorders) were excluded.

Selection Criteria

Study designs included in this review were randomized controlled trials, cross-sectional studies, observational studies, cohort prospective/retrospective studies, and case reports/case series. The physiological mechanisms of skin and wound healing were included, with a primary focus on how the effects of MN play a significant role in these processes. Studies that explored the therapeutic role, clinical applications, and recent advancements in MN were also included. Literature reviews, systematic reviews, scoping reviews, abstracts, and opinion pieces were excluded from the analysis. Literature that did not focus primarily on how MN can impact skin physiology and skin health was excluded. 

MN effects on skin

**Figure 2:**
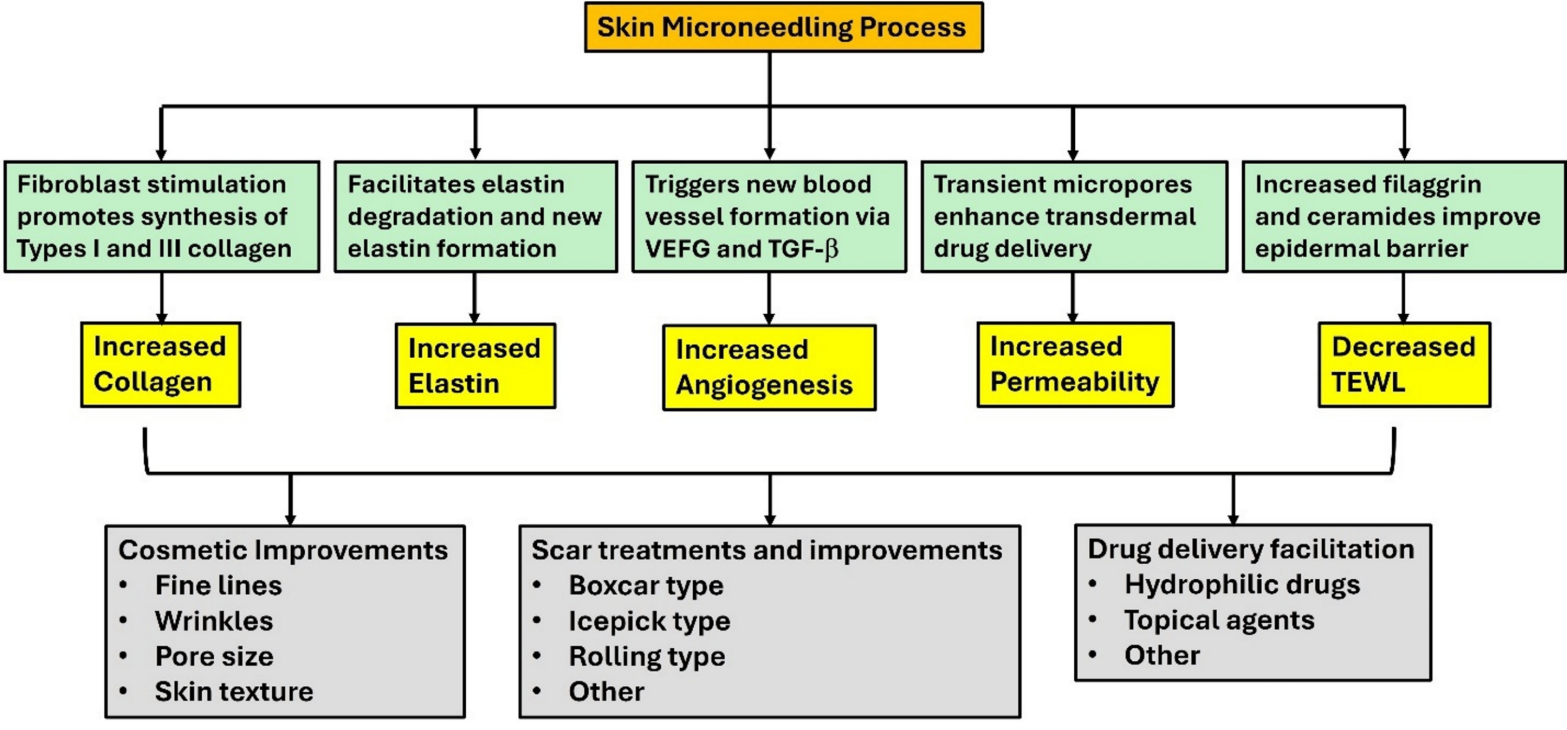
Major physiological processes and treatment targets involved in microneedling TEWL: transepidermal water loss; VEGF: vascular endothelial growth factor; TGF-β: transforming growth factor beta

Figure 2 provides an overview of the major physiological processes involved in the MN process.

Skin Permeability 

Transdermal drug delivery is innately limited due to the inability of large macromolecules to pass the stratum corneum layer. Various methods have evolved to overcome this limitation. These include using chemical enhancers [15], using iontophoresis [16], and using photoacoustic effects created by pressure waves [17]. The MN process increases skin permeability, facilitating drug delivery across the skin barrier and directly into the vascularized dermis by creating microchannels, a process called "microporation". This increased permeability allows the transport of hydrophilic water-soluble molecules to enhance drug delivery [18]. These micropores can remain open anywhere from a few minutes to several hours, depending on the depth of the MN treatment, the individual's skin characteristics, and the type of post-needling treatment being applied [19]. Although increased skin permeability from these micropores can facilitate transdermal drug delivery, improve absorption of therapeutic agents, and stimulate angiogenesis, it can also lead to a compromised skin barrier that is more susceptible to irritation, infection, and post-inflammatory hyperpigmentation [3]. Most side effects tend to be transient, with bruising and edema being immediate and lasting 3-4 days [20].

Collagen Induction 

Skin collagen and elastin are two structural proteins that contribute to the firmness and elasticity of the skin. Predominating in infancy, these proteins progressively decline with aging [21]. The aging process is further accelerated by the natural decline in activity in fibroblasts, the central collagen-synthesizing cells of the skin. This is particularly evident among women during the first five years of menopause, as they experience a loss of 30% of skin collagen, which can dramatically affect skin texture and quality [22]. Collagen fibers are responsible for the skin's tensile strength and compression resistance [23]. MN enhances these innate properties of the skin by stimulating collagen induction through wound healing facilitated by fibroblasts. Following the first phase of MN-induced wound healing, fibroblasts migrate to the wounded area and produce type III collagen, elastin, glycosaminoglycans, and proteoglycans. Type III collagen is continuously converted to type I collagen via collagenases and matrix proteinases, contributing to the firmness of the healed epidermis [24]. In a study analyzing the effects of MN with topical growth factors, researchers found that adding these growth factors augments collagen remodeling [25]. Furthermore, researchers found that four months after patients were treated with fractional radiofrequency MN, histopathologic analysis of the biopsies revealed an increase in the density and compaction of collagen fibers. Additionally, it was reported that each patient displayed a 30% average reduction in fine lines and wrinkles in the lower face and neck [26].

Elastin Production

Elastic fibers are composed of elastin and microfibrils, contributing to the skin's stretchability and recoil effectiveness. The main component of elastin is tropoelastin, which gradually decreases with skin aging [27]. In a study investigating the effects of MN on atrophic acne scars, a statistically significant decrease (p<0.05) in total elastin was reported one month post-treatment along with a contrasting statistically significant increase (p<0.05) in collagen types I, III, and VII. This initial elastin decrease is hypothesized to be due to the accelerated degradation of existing elastin as a precursor to new elastin synthesis. However, elastin formation increased three months after the initial MN treatment [28].

Angiogenesis 

MN promotes new blood vessel formation (angiogenesis). The initial MN-induced injury triggers the release of multiple growth factors, including vascular endothelial growth factor (VEGF), PDGF, transforming growth factor beta (TGF-β), epidermal growth factor (EGF), and connective tissue growth factor (CTGF) [29]. PDGF and TGF are secreted by monocytes and stimulate epithelization, angiogenesis, and collagen production [30]. Among these growth factors, VEGF plays a critical role in angiogenesis. It is associated with increased blood circulation, maturing blood vessels, and enhancing blood vessel perfusion by promoting vasodilation and attracting migrating cells [30]. MN therefore facilitates the release of pro-angiogenic growth factors which work to increase blood circulation and prevent hypoxic damage by enhancing tissue repair. Such MN-induced angiogenesis has been reported to promote the viability of skin flaps in an experimental animal model [31].

Epidermal Barrier Enhancement 

The skin is the largest organ in our body, playing a critical role as a protective barrier [32]. The stratum corneum is the most superficial layer, and its function is maintained through its water-holding capacity and hydration. The stratum corneum contains corneocytes surrounded by a matrix of specialized lipids [33]. The stimulation of collagen and elastin helps restore and strengthen the barrier following injury. MN capitalizes on this process by creating a controlled injury. A study that used six MN treatments performed at two-week intervals reported skin-enhancing and rejuvenation effects that included increased epidermal thickness and increased levels of tropoelastin, collagen types I, III, and VII, as well as newly formed collagen [34]. Changes in the epidermal and dermal layers of the skin were measured at baseline, one month, and three months of treatment, revealing statistically significant enhancements at both one month and three months following MN sessions compared to baseline. 

Filaggrin as a MN target 

Physiological Role of Filaggrin 

Proper skin barrier function primarily depends on the structure and composition of the stratum corneum layer of the epidermis, which comprises corneocytes surrounded by an organized lipid matrix [35]. The corneocytes, essentially called the "bricks" of the stratum corneum layer, are flattened, anucleated cells that consist of filaggrin-aggregated keratin bundles [36]. Filaggrin is essential to the epidermal barrier's structure and function. It starts as a profilaggrin polymer in the stratum granulosum before being dephosphorylated into filaggrin monomers in the stratum corneum [37]. Then, transglutaminases cross-link filaggrin proteins with keratin and other proteins, such as loricrin and involucrin, to form an envelope surrounding corneocytes [38]. This cornified envelope of proteins anchors corneocytes to the highly organized extracellular lipid matrix, forming the epidermal barrier. Filaggrin is crucial for adequately developing the epidermal structure and barrier function. It contributes to the physical structure of corneocytes and indirectly aids in moisturization via its degradation products. This protein is ultimately degraded into histidine and other amino acid metabolites such as urocanic acid and pyrrolidine carboxylic acid, also referred to as natural moisturizing factors (NMFs) [39].

These filaggrin degradation products play a role in the water-holding capacity and hydration of the stratum corneum and are crucial for epidermal barrier homeostasis [39]. A filaggrin deficiency can disrupt the stratum corneum's moisture-retaining capacity and hydration by disrupting corneocyte structure and diminishing the production of NMF. Many skin barrier disorders, such as atopic dermatitis, ichthyosis vulgaris, and xerosis cutis, have been associated with filaggrin deficiency. A study analyzing filaggrin knockdown in human differentiated keratinocytes showed that filaggrin loss-of-function (LOF) mutations are strongly associated with atopic dermatitis. Moreover, the discovery of LOF mutations in filaggrin was shown to cause ichthyosis vulgaris [37]. Filaggrin degradation products, namely, histidine, urocanic acid, and pyrrolidone-5-carboxylic acid, also contribute to the acidification of the stratum corneum [40]. These factors form ceramides, a group of lipids involved in skin barrier structure and maintenance. A deficiency in filaggrin has been shown to disrupt the order of the intercellular lipid matrix, thus affecting the barrier function [41]. Another finding revealed that individuals with atopic skin disorders, such as eczema, cannot produce sufficient amounts of ceramides and many exhibit LOF gene mutations in filaggrin protein.

MN Impacts on Filaggrin 

MN has been shown to increase filaggrin areas by enhancing wound repair and increasing skin cell turnover and strength. By inducing micro-injuries to the skin, the wound healing process is initiated with an increase in various growth factors and cytokines, keratinocyte proliferation, and upregulation of transglutaminase-1 (TGM-1), a key cross-linker of filaggrin as discussed previously [42]. In a human ex vivo human tissue model study, skin barrier function and epidermal regeneration were assessed by taking 10 human donor skin samples, subjecting them to MN treatment, culturing them for six days, and then analyzing the tissue via the expression of factors ki67 (a marker of cell proliferation), filaggrin, and TGM-1. Expression of factors ki67, filaggrin, and TGM-1 was measured on day 0 (immediately post-MN treatment) and on day 6 (after six days of culture). The study revealed a statistically significant increase in the expression of the aforementioned factors on day 6 compared to day 0, demonstrating that MN enhances filaggrin and other skin barrier biomarkers, as well as pro-angiogenic factors. It is a process that requires several days for recovery, including the initiation of the wound healing process, enhanced keratinocyte proliferation, improved skin barrier function, and strengthening of the cornified envelope following MN treatment [43]. Another study reported that post-treatment of MN applied to human full-thickness skin models with dexpanthenol ointment, an analog of D-pantothenic acid (vitamin B5), significantly accelerated wound healing. It was concluded that only in the presence of topical dexpanthenol was MN able to upregulate filaggrin [44]. Further investigations are needed to quantify MN's effects on filaggrin and its role in the skin's regenerative behavior.

MN impacts on ceramides

Ceramides are structural components of the lipid barrier and directly influence the skin's ability to retain moisture. These molecules are essential for the stratum corneum's lipid layer and comprise 40-50% of the intercellular lipid membrane [45]. Comprised of corneocytes, the stratum corneum is continuous with this lipid matrix. The organization of the stratum corneum with the intercellular matrix also plays a role in maintaining the moisture and function of the epidermal barrier. This highly organized lipid membrane is composed of cholesterol, free fatty acids, and ceramides [46]. Ceramides form a permeability barrier that helps prevent excessive water loss [41]. Similar to the effects of reduced levels of filaggrin, decreased ceramide content in the stratum corneum is associated with defective barrier function. It can lead to numerous skin disorders, such as atopic dermatitis [46]. As such, a ceramide-containing moisturizer has been reported to significantly improve skin barrier function in patients with atopic dermatitis [47].

A 12-week study was conducted on 28 individuals undergoing MN treatment for skin aging [48]. It was determined that MN with a solution containing human adipose tissue stem cell-derived exosomes (ASCEs) significantly improved skin aging. This study concluded that MN, in combination with penetration of the ASCE solution into the dermis, can lead to skin tightening and wrinkle reduction through ceramide induction. Specifically, these ASCEs induce the synthesis of ceramides in the skin, leading to the production of epidermal lamellar bodies and a reduction in transepidermal water loss (TEWL). Specifically, epidermal lamellar bodies secrete lipids and enzymes to maintain skin barrier function and prevent water loss, improving stratum corneum hydration and epidermal barrier integrity. The possible correlation between MN and ceramides has not been studied in humans. However, an in vitro study demonstrated that non-invasive microneedle application can improve skin barrier integrity and moisture by increasing ceramide levels [49]. Using reconstructed human full-thickness skin models, microneedles were applied at loads of 1, 2, or 3 N for 24 hours. TEWL and mRNA expression of inflammatory cytokines were measured to see if the microneedle load damaged the skin barrier. Microneedle application at 1 N load caused no increase in TEWL or inflammatory cytokines, serving as a non-invasive MN model. MN-treated tissue demonstrated an upregulated gene expression of serine palmitoyltransferase long chain base subunit 3 (SPTLC3), the primary enzyme involved in synthesizing the basic skeleton of ceramide. In this study, the MN also increased ceramide concentration; specifically esterified omega-hydroxy sphingosine ceramide (Cer EOS), non-hydroxy sphingosine ceramide (Cer NS), and non-hydroxy phytosphingosine ceramide (Cer NP) all significantly increased relative to control tissues (p<0.05). In addition, the mRNA expression of filaggrin-degrading enzymes, such as caspase 14 and bleomycin hydrolase, significantly increased following the MN of the tissue. Filaggrin breakdown was also reported to cause an increase in NMFs such as phenylalanine, histidine, and tryptophan [49].

TEWL

TEWL measures the amount of water the skin loses and depends on the integrity of the skin's barrier function. MN has been found to temporarily disrupt the skin barrier before initiating the skin's self-healing process. However, in 20 women, six bi-weekly fractional radiofrequency MN treatments decreased TEWL after treatment completion [50]. The TEWL reduction was attributed to increased epidermal and dermal density and diameter thickness, which was measured by collagen content using skin ultrasonography. In a study investigating the histopathological changes of various needle lengths and repair time of TEWL, longer needle lengths of 1.5 mm led to higher average mean TEWL values in comparison to shorter needle lengths of 0.5 mm [51]. Another group of researchers evaluated the transport of various compounds through the skin using microneedle arrays created from hyaluronic acid. The microneedle arrays initially led to elevated TEWL values immediately after treatment before decreasing 90 minutes later.

The temporary increase in TEWL led to increased skin permeability which allowed for the facilitation and transport of molecules across the skin [52]. This study revealed that MN could effectively enhance drug delivery while maintaining skin safety, as indicated by reversible changes in TEWL following microneedle application. Furthermore, a group of researchers also investigated the skin penetration efficiency of dermarollers to enhance drug delivery [53]. This disruption of the skin barrier, as seen through a temporary increase in TEWL over the first hour, allowed for greater delivery of hydrophilic model drugs into the deeper skin layers.

Cosmetic applications 

One cosmetic-related target of MN is the reduction in the appearance of fine lines, wrinkles, and scars [13]. In a study, 40 persons (20 men and 20 women) aged 33-60 years old were divided into two groups: those who had facial wrinkles due to skin aging or cigarette smoking. The use of the Dermapen was reported to result in a statistically significant improvement in skin texture and tightness in all patients of the skin aging group [54]. In another study, researchers found that after four monthly procedures with an MN pen, patients had a substantial reduction in the appearance of wrinkles 30 days post-treatment, most prominently in the cheeks, marionette lines, glabellar folds, and forehead, respectively. Significant improvements to these signs of facial aging were confirmed by profilometry which is used to characterize skin topography by wrinkle depth and texture [55]. In one additional study, 35 patients received one MN facial and neck every four months which was reported to increase facial elasticity and dermal and epidermal density when measured three months post-treatment [56]. In this study, elasticity was measured using an infrared laser device which can quantify skin changes in a vacuum-sealed environment.

Treating skin scars 

MN has been found to aid in the treatment of some atrophic facial acne scars [57]. Atrophic scars can be categorized into three subtypes according to their appearance and morphology: box scars, icepick scars, and rolling scars [58]. Icepick scars are narrow (<2 mm) and extend vertically, tapering into the deep dermis. Rolling scars are generally 4-5 mm wide with sloped and shallow borders, and boxcar scars are 1.5-4 mm wide with round to oval depressions and vertical edges [59]. In a study evaluating the effectiveness of the Dermaroller on acne scars, researchers found that icepick and boxcar scars were more responsive to treatment than rolling scars, with a noticeable change in the size and depth of box scars [60]. Another study involving 120 patients with various facial and non-facial scars received one to six consecutive MN treatments. After an average of 2.5 treatments, all scars improved by 50%, with 80% of patients having an improvement between 50% and 75% and 65% of patients having an improvement of over 75% [61]. The degree of scar improvement was measured by two trained medical assistants who utilized the five-point Global Assessment Score (0=no change, 1=1-25% improvement, 2=26-50% improvement, 3=51-75% improvement, 4=76-100% improvement) when evaluating patient photographs. Another study compared MN to carboxytherapy (CXT) for treating atrophic post-acne scars [62]. CXT is a treatment consisting of gaseous carbon dioxide injections into cutaneous and subcutaneous tissue. The study result indicated a significant decrease in all types of scars (icepick, boxcar, and rolling) (p≤0.001). Thirty-two patients with atrophic scars received six sessions of MN on the left side of the face and CXT on the right side of the face. Both treatments resulted in improved organization of collagen and elastic fibers and increased epidermal thickness with no significant difference between the two (p>0.05).

Transdermal drug delivery

Transdermal drug delivery is helpful in directly targeting pathologic sites on the skin, avoiding systemic adverse reactions in patients, and improving drug bioavailability by avoiding first-pass hepatic metabolism [63]. Therefore, a key aspect of transdermal drug delivery research is to find minimally invasive approaches that increase skin permeability without losing skin vitality and function in the long term. MN is a viable solution in enhancing transdermal drug delivery, as this procedure is minimally invasive while having the ability to compromise the stratum corneum barrier and create microchannels that can effectively deliver drugs to the skin [64]. Transdermal drug needling via MN procedures has been shown to deliver drugs more efficiently by delivering drugs directly into the skin layers, enhancing drug bioavailability, and improving therapeutic efficacy [65]. By creating non-permanent microchannels and initiating the wound-healing process, MN provides a minimally invasive procedure that facilitates drug delivery while improving skin texture and vitality at the same time. The literature indicates that MN statistically improves transdermal drug delivery methods, which could be applied to various applications such as cosmetics, skin disease, and vaccine delivery [66]. One study assessed the transdermal delivery of acyclovir, a viral drug, using solid silicon microneedles of varying lengths and densities to pretreat human cadaver skin. The study found that microneedles greater than 0.6 mm in length led to a statistically significant increase in acyclovir flux across the skin compared to shorter needle lengths in the range of 0.1 mm, 0.2 mm, and 0.3 mm [67]. Additionally, arrays with a lower needle density (<2000 needles/cm^2^) were more effective in enhancing acyclovir flux. These findings suggest that skin permeability alterations may most effectively control transdermal drug transport. Several researchers analyzed the optimal routes for how various MN delivery methods can enhance this drug transport by creating an algorithm that examined the effects of microneedle length, radius, spacing, and densities [68]. This study reported that the optimal surface area and radius depend on the type of MN being used. Solid microneedles work optimally at a 0.065 mm radius, while hollow microneedles work best at a 0.145 mm radius. Lower micropore densities statistically improved drug delivery independent of the type of MN formulation used. Another study used solid, coated, hollow, and dissolving needles for MN procedures [69]. They found that the efficacy of each microneedle array type depended on the drug formulation and desired therapeutic effect. Solid microneedles were reported as effective in delivering peptide formulations. In contrast, coated microneedles were reported to enhance local drug delivery, while hollow microneedles were more suitable for continuous drug delivery formulations. Dissolving microneedles was reported to be more effective in systemic therapies such as delivering antibiotics. As such, MN appears to be a helpful tool in optimizing transdermal drug delivery. This can be useful both in cosmetics and in the study of future drug therapies for treating diseased states.

Safety considerations and side effects

As with all modes of dermatologic treatments, the safety considerations and side effects of MN must be assessed. However, rare and mild adverse effects can depend on the type of MN device used. Among various MN modalities, erythema was found to be statistically significant with the stamp modality followed by the pen, roller, and radiofrequency energy [70]. Edema was statistically significant with the roller [71], while pain was more significant with the pen [72]. Lastly, tram-tracking and post-inflammatory hyperpigmentation were more significant with the roller [73], while bleeding was most significant with the roller [74]. As future studies are encountered, it is critical to assess the safety profile of MN for potential complications, including granulomatous reactions and local and systemic hypersensitivity reactions to both instrumentation and topicals used in treatment [3]. Some conclusions on the impacts of MN on dermatologic conditions have been reported based on case studies and small-scale randomized controlled trials [75].

## Conclusions

MN is a minimally invasive technique that creates micro-injuries to the epidermis and dermis that stimulate the skin's intrinsic wound repair cascade. This review evaluated and summarized MN's physiologic mechanisms and therapeutic applications. The main physiological mechanisms associated with the MN process were collagen and elastin production, angiogenesis, transient increases in skin permeability, and improved post-treatment epidermal barrier function. However, no information on direct measurements of the skin's biophysical changes, such as altered hydration status or its in vivo mechanical properties post-MN treatment, was systematically reported. Therapeutic applications targeted cosmetic improvements, scar healing, and drug delivery. Key processes involved were related to the wound repair process in which fibroblasts migrate to the wounded area to initiate collagen and elastin production, contributing to the improved firmness and elasticity of the healed epidermis. The micropores increase skin permeability, allowing hydrophilic water-soluble molecules to transfer across the skin to enhance transdermal drug delivery and absorption. Multiple growth factors, such as VEGF and TGF-β, are secreted by monocytes upon injury and contribute to collagen production, epithelization, and angiogenesis that increase epidermal thickness and contribute to the epidermal barrier enhancement found post-procedure. Additionally, TGFM-1, a cross-linker of the protein filaggrin, and ki67, a marker of cell proliferation, are upregulated following the controlled tissue injury. These upregulated biomarkers contribute to the increase in filaggrin, enhancing skin barrier function. Ceramides, which help retain moisture and prevent TEWL, are also increased when MN is combined with a solution containing human ASCEs.
